# Prevalence of pathogenic/likely pathogenic variants in the 24 cancer genes of the ACMG Secondary Findings v2.0 list in a large cancer cohort and ethnicity-matched controls

**DOI:** 10.1186/s13073-018-0607-5

**Published:** 2018-12-24

**Authors:** Jung Kim, Wen Luo, Mingyi Wang, Talia Wegman-Ostrosky, Megan N. Frone, Jennifer J. Johnston, Michael L. Nickerson, Melissa Rotunno, Shengchao A. Li, Maria I. Achatz, Seth A. Brodie, Michael Dean, Kelvin C. de Andrade, Fernanda P. Fortes, Matthew Gianferante, Payal Khincha, Mary L. McMaster, Lisa J. McReynolds, Alexander Pemov, Maisa Pinheiro, Karina M. Santiago, Blanche P. Alter, Neil E. Caporaso, Shahinaz M. Gadalla, Lynn R. Goldin, Mark H. Greene, Jennifer Loud, Xiaohong R. Yang, Neal D. Freedman, Susan M. Gapstur, Mia M. Gaudet, Donato Calista, Paola Ghiorzo, Maria Concetta Fargnoli, Eduardo Nagore, Ketty Peris, Susana Puig, Maria Teresa Landi, Belynda Hicks, Bin Zhu, Jia Liu, Joshua N. Sampson, Stephen J. Chanock, Lisa J. Mirabello, Lindsay M. Morton, Leslie G. Biesecker, Margaret A. Tucker, Sharon A. Savage, Alisa M. Goldstein, Douglas R. Stewart

**Affiliations:** 1 Clinical Genetics Branch, Division of Cancer Epidemiology and Genetics, National Cancer Institute, NIH, Rockville, MD 20850 USA; 2 Cancer Genomics Research Laboratory, Division of Cancer Epidemiology and Genetics, National Cancer Institute, Leidos Biomedical Research, Inc., Frederick, MD 21701 USA; 3 División de Investigación, Instituto Nacional de Cancerología, 14080 Mexico City, Mexico; 4 Medical Genomics and Metabolic Genetics Branch, National Human Genome Research Institute, NIH, Bethesda, MD 20892 USA; 5 Laboratory of Translational Genomics, Division of Cancer Epidemiology and Genetics, National Cancer Institute, NIH, Gaithersburg, MD 20877 USA; 6 Epidemiology and Genomics Research Program, Division of Cancer Control and Population Sciences, National Cancer Institute, NIH, Rockville, MD 20850 USA; 7 International Research Center, A.C. Camargo Cancer Center, São Paulo, 01508-010 Brazil; 8 Occupational and Environmental Epidemiology Branch, Division of Cancer Epidemiology and Genetics, National Cancer Institute, NIH, Rockville, MD 20850 USA; 9 Integrative Tumor Epidemiology Branch, Division of Cancer Epidemiology and Genetics, National Cancer Institute, NIH, Rockville, MD 20850 USA; 10 Metabolic Epidemiology Branch, Division of Cancer Epidemiology and Genetics, National Cancer Institute, NIH, Rockville, MD 20850 USA; 11 Behavioral and Epidemiology Research Group, American Cancer Society, Atlanta, GA USA; 12 Department of Dermatology, Maurizio Bufalini Hospital, Cesena, Italy; 13 Department of Internal Medicine and Medical Specialties, University of Genoa and Genetics of Rare Cancers, IRCCS Ospedale Policinico San Martino, Genoa, Italy; 14 Department of Dermatology, University of L’Aquila, L’Aquila, Italy; 15 Department of Dermatology, Instituto Valenciano de Oncologia, Valencia, Spain; 16 Institute of Dermatology, Catholic University - Fondazione Policlinico Universitario A. Gemelli, IRCCS, Rome, Italy; 17 Dermatology Department, Melanoma Unit, Hospital Clinic de Barcelona, IDIBAPS, Universitat de Barcelona, Barcelona, Spain & Centro de Investigacion Biomedica en Red en Enfermedades Raras (CIBERER), Valencia, Spain; 18 Biostatistics Branch, Division of Cancer Epidemiology and Genetics, National Cancer Institute, NIH, Rockville, MD 20850 USA; 19 Office of the Director, Division of Cancer Epidemiology and Genetics, National Cancer Institute, NIH, Rockville, MD 20850 USA; 20 Radiation Epidemiology Branch, Division of Cancer Epidemiology and Genetics, National Cancer Institute, NIH, Rockville, MD 20850 USA; 21 Division of Cancer Epidemiology and Genetics, Human Genetics Program National Cancer Institute, NIH, Rockville, MD 20850 USA; 22 Centro de Oncologia, Hospital Sirio-Libanes, Sao Paulo, SP 013050-050 Brazil

**Keywords:** ACMG secondary findings, Familial cancer exome, Population study, Variant classification

## Abstract

**Background:**

Prior research has established that the prevalence of pathogenic/likely pathogenic (P/LP) variants across all of the American College of Medical Genetics (ACMG) Secondary Findings (SF) genes is approximately 0.8–5%. We investigated the prevalence of P/LP variants in the 24 ACMG SF v2.0 *cancer* genes in a family-based cancer research cohort (*n* = 1173) and in cancer-free ethnicity-matched controls (*n* = 982).

**Methods:**

We used InterVar to classify variants and subsequently conducted a manual review to further examine variants of unknown significance (VUS).

**Results:**

In the 24 genes on the ACMG SF v2.0 list associated with a *cancer* phenotype, we observed 8 P/LP unique variants (8 individuals; 0.8%) in controls and 11 P/LP unique variants (14 individuals; 1.2%) in cases, a non-significant difference. We reviewed 115 VUS. The median estimated per-variant review time required was 30 min; the first variant within a gene took significantly (*p* = 0.0009) longer to review (median = 60 min) compared with subsequent variants (median = 30 min). The concordance rate was 83.3% for the variants examined by two reviewers.

**Conclusion:**

The 115 VUS required database and literature review, a time- and labor-intensive process hampered by the difficulty in interpreting conflicting P/LP determinations. By rigorously investigating the 24 ACMG SF v2.0 *cancer* genes, our work establishes a benchmark P/LP variant prevalence rate in a familial cancer cohort and controls.

**Electronic supplementary material:**

The online version of this article (10.1186/s13073-018-0607-5) contains supplementary material, which is available to authorized users.

## Background

In 2013, the American College of Medical Genetics and Genomics (ACMG) recommended that “laboratories performing clinical [exome or genome] sequencing seek and report mutations of the specified classes or types” in a set of 56 genes associated with a severe phenotype, and for which disease risk may be reduced or managed before symptoms arise [1, 2]. These recommendations for reporting of incidental (or secondary) findings (SF) in clinical exome and genome sequencing were later amended to 59 genes (ACMG SF v2.0) [3].

Although both ACMG SF policy statements used the older “known pathogenic” or “expected pathogenic” variant categorization terminology [4], a transition to the newer five-category system of pathogenicity has been urged [5, 6]. To date, multiple studies using the newer pathogenicity scheme to investigate clinical exome sequencing data and publicly available sequence databases in primarily European-American and African-American cohorts have estimated the prevalence of ACMG SF gene list (the original 2013 list and 2017 amendment) pathogenic/likely pathogenic (P/LP) variants to be approximately 0.8–5% [7, 8]. Some, but not all, studies of ethnically diverse cohorts have found higher prevalence of P/LP (5.6–7%) for ACMG SF genes [9–11]. The prevalence of P/LP variants in *cancer* cohorts remains largely uninvestigated, and to our knowledge, prevalence of P/LP variants has not been determined in a large cancer study with ethnicity-matched healthy controls.

The current American College of Medical Genetics and Genomics/Association for Molecular Pathology (ACMG/AMP) guidelines use conservative methods to classify variants based on numerous criteria including clinical and family history, previous literature, and known population allele frequency [5]. Currently, there are 28 criteria used to determine final variant classification, and the use of these criteria is labor-intensive. One strategy for applying the ACMG/AMP guidelines is a consensus-based tumor board-like review by experts for genes/variants of interest. However, this approach is relatively low-throughput, labor-intensive, and not realistic for large-scale sequencing efforts. Another strategy would employ automated procedures. The software package InterVar was developed as a semi-automated approach to applying the ACMG/AMP guidelines [12]. It incorporates 10 of 28 ACMG/AMP criteria automatically; the remaining 18 criteria can be applied following manual review of a variant in the literature, if published.

In this study, we used the most recent ACMG/AMP criteria and determined the prevalence of P/LP variation in the 24 ACMG SF v2.0 gene list associated with a *cancer* phenotype in a large, family-based heterogeneous cancer research cohort (*n* = 1173 individuals; 738 families) and in ethnicity-matched controls (*n* = 982). (The remaining 35 *non-cancer* ACMG SF v2.0 genes were not fully investigated.) We used InterVar to classify variants, followed by a manual review to further examine variants of unknown significance (VUS). In addition, we estimated the time to resolve VUS and evaluated the concordance rate between reviewers for 30% of the reviewed variants.

## Methods

### DCEG familial exome cohort and cancer-free controls, anonymization, and ethics review

Cases were drawn from the NCI Division of Cancer Epidemiology and Genetics (DCEG) Familial Exome cohort, a large, long-term, longitudinal, heterogeneous group of family-based studies with a cancer phenotype and a Mendelian or near-Mendelian pattern of inheritance. The majority of the families lacked a known causative germline genetic variant; the cancer phenotype in the families may or may not overlap with the known cancer phenotype of the 24 ACMG v2.0 cancer genes*.* Families in which a causative gene was identified were not excluded. Data from 982 controls from 2 cohort studies, Prostate, Lung, Colorectal, and Ovarian Cancer Screening Trial (PLCO [13]) and the Cancer Prevention Study (CPSII) of the American Cancer Society [14], and 1 case-control study, the Environment and Genes in Lung Cancer Etiology (EAGLE [15]), were available for inclusion in the current study. Controls were cancer-free at the time of enrollment. Controls in the CPSII and PLCO studies were followed longitudinally, and if cancer developed, this was noted; EAGLE controls were not followed longitudinally. All participants provided written consent and were recruited through IRB-approved protocols. For these analyses, cases and controls underwent irrevocable anonymization. The project was reviewed and approved by the NIH Office of Human Subjects Research Protection, which granted a waiver of the IRB review requirement.

### Exome sequencing, quality control, ethnicity determination, and analysis of population stratification

Exome sequencing was performed at the Cancer Genomics Research Laboratory, National Cancer Institute (CGR, NCI), as described [16, 17]. Cases and controls were matched using an ethnicity-informative variation [18]. After the controls and cases were matched, poor quality and contaminated samples were excluded from the dataset. Any variants that were flagged with our pipeline quality control metric (CScorefilter), had a read depth < 10, ABHet < 0.2 or > 0.8, or did not pass other quality control filters were excluded from the analysis. All variants were further filtered using popmaxfreq < 0.01, see Additional file 1: Supplemental Methods for additional details.

### Automated and manual review of variation in the 24 ACMG SF v2.0 cancer genes

Variation in the 24 ACMG SF v2.0 genes primarily associated with a *cancer* phenotype (“ACMG SF v2.0 cancer”: *APC*, *BMPR1A*, *BRCA1*, *BRCA2*, *MEN1*, *MLH1*, *MSH2*, *MSH6*, *MUTYH*, *NF2*, *PMS2*, *PTEN*, *RB1*, *RET*, *SDHB*, *SDHC*, *SDHD*, *SMAD4*, *STK11*, *TP53*, *TSC1*, *TSC2*, *VHL*, *WT1*) was annotated using ANNOVAR [19], which included InterVar, a semi-automated software tool which applies the ACMG-AMP guidelines [12]. To more fully classify potentially pathogenic variants, all ACMG SF v2.0 *cancer* gene variants listed in the Human Gene Mutation Database (HGMD; version 2015.2; Qiagen, Cardiff, Wales, UK) as “disease mutation” (DM) underwent manual review, regardless of the InterVar assertion and without knowledge of case or control status. In addition, we used Google Scholar to search the published literature for information on variants designated VUS by InterVar which were not listed in HGMD. The primary literature was then reviewed by 17 reviewers (including oncologists, hematologists, clinical geneticists, genetic counselors, geneticists, or genetic epidemiologists). The reviewers were assigned specific gene(s) after variant review training and classified the variants according to the ACMG/AMP guidelines using a pre-populated Excel file that contained needed variant annotation information. Reviewers were also asked to provide comments for each score provided and to estimate the time needed to review each variant. We noted which variant within each gene was the first one evaluated by each reviewer. After initial review, variants were subject to a quality control (QC) process in which the criteria for scoring and reviewer comments were compared for agreement. As a second QC check, 31% (*n* = 36) of the 115 variants initially reviewed were re-evaluated by a second independent reviewer. If there was discordance between the primary and secondary reviewers on variant classification, discussion was initiated to reach consensus. The ACMG/AMP combining criteria were implemented using the Genetic Variant Interpretation Tool available online (http://www.medschool.umaryland.edu/Genetic_Variant_Interpretation_Tool1.html/) [20]. Graph and *p* values (*t* test) were calculated using GraphPad Prism 7 (GraphPad Software Inc., La Jolla, CA), and 95% confidence intervals were calculated using STATA 14 (StataCorp LLC, College Station, TX).

## Results

### Sequence quality, demographics, and matching cases and controls

For the entire DCEG Familial Exome cohort (plus controls), exome sequencing was performed such that 88% of coding sequence from the University of California Santa Cruz (UCSC) human genome (hg) 19 transcripts database had ≥ 15 reads with an average coverage of 61×. After the sample quality control, there were 982 control individuals from the PLCO, EAGLE, and CPSII cohorts and 1173 cases (738 families) from 15 cancer-based studies (Tables 1 and 2, Additional file 2: Table S1). Population stratification for Northern and Western European ancestry (CEU) > 0.80 (Additional file 2: Figure S1) resulted in well-matched cases and controls by principal component analysis (Additional file 2: Figure S2).

**Table 1:** Demographic characteristics of cases

Variable	Cases
Ancestry	European (CEU > 0.80)
Sex	M: 682 (58%) F: 491 (42%)
Number of families	738 families
Number of individuals	1173
Involved studies	See Additional file 2: Table S1

*CEU* Northern and Western European ancestry

**Table 2:** Demographic characteristics of controls

Variable	CPSII	PLCO	EAGLE
Ancestry	European (CEU > 0.80)		
Sex	M: 106 (49%) F: 111 (51%)	M: 217 (59%) F: 153 (41%)	M: 314 (79%) F: 81 (21%)
Number of families	No families		
Number of individuals	217	370	395
Average age (years)	71	67	66
Average follow-up (years)	11.1	9.3	N/A
Number of individuals with a cancer found during follow-up	7	32	Were not followed

*CEU* Northern and Western European ancestry, *CPSII* Cancer Prevention Study II (American Cancer Society), *EAGLE* Environment and Genes in Lung Cancer Etiology, *F* female, *M* male, *N/A* not applicable, *PLCO* Prostate, Lung, Colorectal and Ovarian Cancer Screening Trial

### InterVar classification of ACMG SF v2.0 cancer and non-cancer genes prior to expert review

We used InterVar to classify all filtered variants into 6 categories (pathogenic (P), likely pathogenic (LP), variant of unknown significance (VUS), likely benign (LB), benign (B), and no classification) for cases and controls. Since our cohort includes family members, we performed 2 separate analyses: first, we used all cases, and second, we randomly selected 1 affected individual per family. Table 3 shows the InterVar classification of the variants for *all* ACMG SF v2.0 genes, divided into “cancer genes” and “non-cancer genes” columns. In cancer genes, there were 760 variants deemed VUS or “no classification”; “no classification” variants were primarily intronic, located in the 5′ or 3′ untranslated regions, or indels. There were 8 unique P variants (controls and cases); 2 were in *MUTYH. MUTYH* is the only ACMG SF v2.0 *cancer* gene in which the phenotype is associated with an autosomal recessive pattern of inheritance and is therefore reportable only for compound heterozygotes or homozygotes [21]. Since all subjects in this study harbored only 1 P/LP *MUTYH* variant, we excluded this gene from our prevalence calculation.

**Table 3:** ACMG SF v2.0 genes classified per ACMG/AMP guidelines using InterVar software

Classification	Controls (982 exomes)		All cases (1173 exomes)		1 case/family (738 exomes)	
	Cancer genes	Non-cancer genes	Cancer genes	Non-cancer genes	Cancer genes	Non-cancer genes
Pathogenic	4 (17)	0	4 (13)	0	4 (7)	0
Likely pathogenic	0 (0)	8 (8)	0 (0)	11 (17)	0 (0)	10 (11)
Variant of unknown significance	141 (174)	403 (503)	179 (260)	440 (713)	140 (167)	336 (454)
Likely benign	248 (463)	398 (671)	242 (570)	417 (865)	202 (355)	332 (541)
Benign	12 (42)	30 (208)	12 (106)	28 (266)	11 (69)	26 (167)
No classification*	252 (3477)	832 (8181)	290 (4315)	814 (10393)	225 (2714)	668 (6535)

Numbers represent unique variant count and number in parenthesis represents allele counts

*Majority of the “no classification” variants were intronic, 5′/3′ untranslated regions, and 22 indels

### InterVar classification of ACMG SF v2.0 cancer genes after expert review

Of the InterVar-determined cancer gene VUS (*n* = 297) or “no classification” variants (*n* = 463) in cases and controls, 115 (15%) had been reported previously, as per queries of HGMD and Google Scholar (VUS variants only). A total of 77 variants were classified as “DM” in HGMD, and an additional 38 VUS variants were identified in the searchable published literature queried through Google Scholar. Of the remaining 645 variants, there was little or no additional published or online information available, and therefore, these variants were not further evaluated. After review by 1 cancer expert, 36 (31%) randomly selected variants underwent review by a second cancer expert. The concordance rate between the primary and secondary reviewers for the pathogenicity category of these 36 variants was 83.3%. Discussion between reviewers led to the resolution of the 6 discrepant variants from the 36 re-reviewed variants(16.7%) in this study. Among the 115 variants reviewed, 2 unique variants were promoted to P from VUS and 5 unique variants were promoted to P from “no classification.” Two unique variants were promoted to LP from VUS, and 1 unique variant was promoted to LP from “no classification” (Additional file 3: Table S2).

### Prevalence of P/LP variation in cases and controls and estimated time to review

**Fig. 1:**
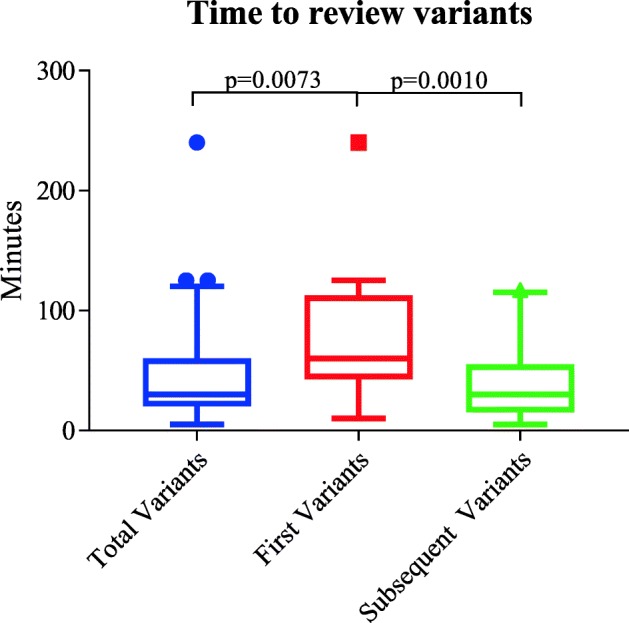
Time required to review variants. Box-and-whisker plot of reviewer-reported per-variant time (in minutes) to conduct manual review of published literature and render a judgment on pathogenicity, as per ACMG/AMP guidelines. There were statistically significant differences in time to review all variants (blue; *n* = 115) vs. the first variant (red; *n* = 24) and first variant vs. subsequent variants (green; *n* = 91)

The allele and total counts of P/LP variants for the 24 ACMG SF v2.0 *cancer* genes after expert review for cases and controls are shown in Table 4. The prevalence of P/LP variants among controls was 0.8% (95% confidence interval (CI) 0.3–1.4%), among cases, 1.2% (95% CI 0.6–1.8%), and for one case per family, 1.1% (95% CI 0.3–1.8%). In controls, the P/LP alleles were in *BRCA2* (five unique), *MSH2* (one), *PMS2* (one), and *TP53* (one) (Additional file 3: Table S2). In cases, the P/LP alleles were in *BRCA1* (one) *BRCA2* (one), *PMS2* (one), and *TP53* (eight unique) (Additional file 3: Table S2). There were no significant differences in the prevalence of P/LP variants between controls and either case set (Table 4). Reviewers needed an estimated median of 30 min (range = 5–240 min) per variant to review the pertinent literature, to consult the ACMG/AMP guidelines, and to make a judgment on the classification criteria (Fig. 1). The first variant examined within a gene took significantly longer (*p* = 0.0009) to review (median = 60 min; range = 10–240 min) compared with subsequent variants in the same gene (median = 30 min; range = 5–117 min). However, these estimated times did not account for the time required to run InterVar, perform a QC check, conduct secondary reviewer validation, and resolve discordances. Incorporating these additional tasks into the review process would result in a much higher time requirement to classify variants.

**Table 4:** Pathogenic and likely pathogenic variants in 24 ACMG SF v2.0 *cancer* genes after expert review

Classification	Controls (982 exomes)	All cases (1173 exomes)	1 case/family (738 exomes)
Pathogenic	6 (6)	5 (6)	3 (3)
Likely pathogenic	2 (2)	6 (8)	4 (5)
Prevalence of pathogenic and likely pathogenic variants	8/982 0.8% 95% CI 0.3–1.4%	14/1173 1.2% 95% CI 0.6–1.8%	8/738 1.1% 95% CI 0.3–1.8%
*p* value (controls vs. all cases)		0.5196	
*p* value (controls vs. 1 case/families)			0.6171

Variants in *MUTYH* were excluded from counts since it underlies a recessive disorder and no *MUTYH* homozygotes or compound heterozygotes were observed in cases or controls. The first number represents unique variant count and number in parenthesis represents allele counts

*CI* confidence interval

## Discussion

In 1173 individuals from a heterogeneous, family-based study of inherited cancer predisposition, the prevalence of P/LP variants in the 24 ACMG SF v2.0 *cancer* genes was 1.2%, not significantly different from P/LP variant prevalence in 982 ethnicity-matched controls (0.8%). Our study is notable for the large cohort size, the use of variation-based ethnicity-matching of cases and controls, thorough expert-driven review of variants by ACMG/AMP criteria, and an exclusive focus on the 24 ACMG SF v2.0 *cancer* genes.

Direct comparison of our results with previous studies is challenging because of the differences in methodology and study populations. We acknowledge that our familial cancer cohort is heterogeneous since it is comprised of individuals drawn from a wide variety of familial tumor-predisposition studies, making comparison difficult. Analyses of the 1000 Genomes and the NHLBI GO Exome Sequencing Project cohorts for P/LP variants in a list of “medically actionable” genes (larger than the ACMG SF v.2.0 list) found a prevalence of 2.2–3.4% [11, 22]. The P/LP prevalence rate (for the original (v1.0) ACMG SF 56-gene list) in smaller, single-institution research cohorts spanned an order of magnitude from 0.86% (Baylor-Hopkins Center for Mendelian Genomics) [23] to 8.8% (Undiagnosed Disease Project) [24].

Data on the prevalence of P/LP variants in cancer cohorts are sparse. To be comprehensive in this research study, we considered both P and LP variants in our analysis, although the threshold to report LP variation from ACMG SF v.2.0 genes in a clinical setting is under debate [6]. One study of 439 individuals undergoing tumor-germline dyad sequencing found that 4.3% harbored a germline variant (in a panel of 247 genes) indicative of hereditary cancer predisposition [25]. A study of 392 patients with pancreatic cancer undergoing tumor/normal sequencing found a prevalence rate of pathogenic variation of 5.1% from a panel of 130 genes [26]. We were not able to find publications that reported prevalence of P/LP for all ACMG SF genes in cancer cohorts. The lower prevalence rate we observed in our study compared with prior publications may be attributable to our evaluating only a subset of known cancer susceptibility genes. In addition, the 24 ACMG SF v2.0 *cancer* genes largely underlie risk in common cancers (e.g., breast, ovarian, and colon cancer) and well-known genetic disorders (e.g., Li-Fraumeni syndrome, retinoblastoma) and are not necessarily associated with the disorders constituting our study cohort (Additional file 2: Table S1). Although one of our studies recruited individuals with a history of familial breast and ovarian cancer, eligibility required documentation of negative germline *BRCA1/2* genetic testing (Additional file 2: Table S1).

The ethnicity-matched controls (PLCO/EAGLE/CPSII) were on average 70 years of age, healthy adults without a history of cancer (other than non-melanoma skin cancer) at the time of study enrollment and sample collection. Interestingly, 0.8% of this control sample harbored a P/LP variant in one of the 24 ACMG SF v2.0 *cancer* genes, not significantly different from the cancer cohort (*p* = 0.5) (Table 4). Furthermore, participants in the CPSII and PLCO study have been followed for an average of 10 years after sample collection. During this follow-up, out of 586 participants from CPSII and PLCO, 39 participants developed cancer. Considering only controls who did not develop cancer after research follow-up, we found a similar prevalence of P/LP variants compared with all controls (1.2% vs. 1.5%, respectively).

By rigorously investigating P/LP variation in the 24 ACMG SF v2.0 *cancer* genes, our work establishes a clinically useful benchmark prevalence rate, especially in controls. Recent studies have shown that pathogenic variation in single genes like *DICER1* [27] and *TP53* [28] (in public datasets like non-TCGA ExAC, 1000G, and ESP) have a higher prevalence than the known or expected population frequency of their associated syndromes. In the case of *DICER1* and *TP53*, the recognition that pathogenic variation in recognized cancer genes is more common than expected is an important, emerging, and unanticipated finding from population-based exome sequencing, one that has significant clinical implications. In this study, we observed P/LP variation in the 24 ACMG SF v2.0 *cancer* genes (specifically, *BRCA2*, *MLH1*, *MSH2*, *PMS2*, and *TP53*) in 0.8% of our 982 controls, who, by a mean age of ~ 70 years, had not developed any malignancy. Thus, in our controls, the prevalence of P/LP germline variation in *BRCA2* was 0.5% (all subjects 5/982, females only 1/345; none were common Ashkenazi variants). In Lynch syndrome genes, the prevalence was 0.4% (*MLH1*, *MSH2*, *PMS2*; excluding *MUTYH* 4/982), and for Li-Fraumeni syndrome, it was 0.1% (*TP53*; 1/982). These frequencies are comparable to other published estimates (*BRCA2* 0.45% in cancer-free Australian women [29]; 0.31% in women of European non-Finnish descent in the Exome Aggregation Consortium, excluding The Cancer Genome Atlas data [30]; Lynch 0.2% [31]). We acknowledge that our controls may not be representative of the entire general population since, as volunteers, the controls may have an interest in cancer studies perhaps due to a family history of cancer.

Reviewers were required to track the estimated amount of time needed to classify variants. Our study is the first to distinguish between the amount of time to review first and subsequent variants within a gene. We found that the first variant took significantly longer to review when compared with subsequent variants, a reflection of the learning curve inherent in applying these new, complex classification algorithms. Although our team was composed of cancer experts, they were not necessarily experts on the specific genes they were reviewing. This could potentially have led to the additional time for familiarization with the gene(s) to be reviewed. Our overall finding that variant review was time-consuming is consistent with previous studies [32]. However, in some clinical labs, a more sophisticated automated pipeline and highly trained variant specialists would likely result in shorter review times. We note that our measurements reflect only the estimated time to review the primary literature and do not include the time required to conduct InterVar classification, secondary review, and consensus-seeking or summation of the ACMG/AMP scores. Since the 24 ACMG SF v2.0 *cancer* genes are recognized and generally well-studied, the amount of available literature (and time spent reviewing it) may be greater than for lesser-known (non-ACMG SF v2.0) genes. In addition, our study population was restricted to people of non-Finnish European ancestry. Published work has highlighted the additional challenges in interpreting genetic variation in non-European populations [33]. Thus, our variant interpretation times may have been shorter compared with those of non-European populations.

Our experience with InterVar and the ACMG/AMP guidelines deserves a brief comment. We found that InterVar was a useful tool to start the initial variant classification using the ACMG/AMP guidelines. Despite the use of InterVar and manual review, most variants remain unresolved due to the lack of published literature and for our study and limited clinical information. Proper classification of variants, especially those used in clinical decision-making, is a time-consuming and laborious process that, for now, requires human expertise and judgment. Currently, this process is more subjective, and yields less reproducible results, than is optimal. In the future, this may be streamlined with more extensive, comprehensive electronic databases of definitively classified variants, more sophisticated software (e.g., neural networks) [34], and artificial intelligence programs (e.g., machine learning) [35], based on formal, probabilistic frameworks [36].

Reviewers in this study frequently noted that gaining a working familiarity with the ACMG/AMP guidelines was demanding. As a quality control procedure, we compared criterion scores (0 or 1) with the respective comments provided by the reviewer; we observed confusion related to multiple criteria. Despite these challenges and after correction of inconsistently scored criteria (based on the comments provided), our secondary review and consensus process showed a concordance rate of 83.3%, which is at the upper limit of previously reported concordance rates (34–79%) [11, 23, 37, 38]. In many cases, ambiguous words in the ACMG/AMP criteria such as “well-established” (e.g., in criteria PM1, PS3, BS3) and “multiple” (PP1, BP4) are subjective and unavoidably led to discrepancies between reviewers in the consensus process. These differences in criteria interpretation were resolvable with a discussion between the primary and secondary reviewers. Suggestions to refine the wording of the ACMG/AMP guidelines, as well as other practical improvements (e.g., specific cutoff MAF for each disease, resources for which genes cause disease by loss of function, which functional assays are appropriate, and the quantitative threshold for segregation) have been promulgated [37]. To resolve this ambiguity, the Clinical Genome Resource (ClinGen) [39] is working with experts in the field to refine the guidelines. For hereditary cancer, there are five different working groups (breast and ovarian cancer, *CDH1*, colon cancer, *PTEN*, and *TP53)*.

We acknowledge the limitations of our study. Since cases and controls were anonymized, there were restrictions on the depth and detail of clinical information. Thus, we were not able to assess de novo or *cis*/*trans* status or to assess segregation of a variant with phenotype. Availability of these data may have increased the number of variants that were definitively classifiable, which would have reduced the number of VUS. In addition, we did not review all VUS variants called by InterVar; we only considered the 115 variants that were reported by HGMD as DM or for which sufficient information was found in Google Scholar. Furthermore, this study only examined the white population, potentially limiting the applicability of these findings on P/LP prevalence to other ethnic groups and variant review time. Lastly, although the cases were drawn from a heterogeneous, convenient cohort of families assembled over multiple decades and protocols, the generalizability of our results may be limited, given the broad spectrum of cancer diagnoses.

## Conclusions

We found a non-significant difference in the prevalence of P/LP variants from the ACMG SF v2.0 *cancer* genes in a cancer cohort (1.2%) and ethnically matched healthy controls (0.8%). Variant review, even with the help of sophisticated software tools, is time-consuming. Newer approaches, perhaps using artificial intelligence tools and neural networks, are needed to simplify and expedite this important task.

## Additional files

Additional file 1: Supplemental Methods. Detailed methods on DNA preparation, hybridization, exome sequencing, variant calling, use of ethnicity-informative variation, quality control, and capture region matching. (PDF 233 kb)

Additional file 2: **Figure S1.** Population stratification of cancer cases and controls. **Figure S2.** Principal component analysis of cancer cases and controls. **Table S1.** Study names and predominant cancer types in DCEG Familial Exome cohort. (PDF 449 kb)

Additional file 3: **Table S2.** InterVar classification of ACMG SF v2.0 cancer genes after expert review. (XLSX 30 kb)

## Abbreviations

ACMG: American College of Medical Genetics and Genomics
ACMG/AMP: American College of Medical Genetics and Genomics/Association for Molecular Pathology
B: Benign
CEU: Northern and Western European ancestry
CGR, NCI: Cancer Genomics Research Laboratory, National Cancer Institute
CPISII: Cancer Prevention Study
DCEG: Division of Cancer Epidemiology and Genetics
DM: Disease mutation
EAGLE: Environment and Genes in Lung Cancer Etiology
HGMD: Human Gene Mutation Database
LB: Likely benign
LP: Likely pathogenic
P: Pathogenic
P/LP: Pathogenic/likely pathogenic
PLCO: Prostate, Lung, Colorectal, and Ovarian Cancer Screening Trial
QC: Quality control
SF: Secondary finding
VUS: Variants of unknown significance
