# Supplementary material for: Prevalence of pathogenic/likely pathogenic variants in the 24 cancer genes of the ACMG Secondary Findings v2.0 list in a large cancer cohort and ethnicity-matched controls
Source: Genome Med. 2018 Dec 24;10:99. doi: 10.1186/s13073-018-0607-5 (PMC6305568; doi:10.1186/s13073-018-0607-5)
Supplement: Supplementary file 1 — Supplemental Methods. Detailed methods on DNA preparation, hybridization, exome sequencing, variant calling, use of ethnicity-informative variation, quality control, and capture region matching. (PDF 233 kb) [file 13073_2018_607_MOESM1_ESM.pdf]

Supplemental Methods

DNA preparation. For each sample, 200 ng genomic DNA was purified using Agencourt AMPure XP Reagent (Beckman Coulter Inc, Brea, CA, USA) according to the manufacturer's protocol. An adapter-ligated library was prepared with the KAPA HyperPlus Kit (KAPA Biosystems, Wilmington, MA) using Bioo Scientific NEXTflex™ DNA Barcoded Adapters (Bioo Scientific, Austin, TX, USA) according to the KAPA-provided protocol.

Pre-hybridization LM-PCR. Genomic DNA sample libraries were amplified pre-hybridization by ligation-mediated PCR consisting of one reaction containing 20 µL library DNA, 25 µL 2x KAPA HiFi HotStart ReadyMix, and 5µL 10x Library Amplification Primer Mix (includes two primers whose sequences are: 5'-AATGATACGGCGACCACCGA-3' and 5'-CAAGCAGAAGACGGCATACGA-3'). PCR cycling conditions were as follows: 98˚C for 45 seconds, followed by 5 cycles of 98˚C for 15 s, 60˚C for 30 s, 72˚C for 30 s. The last step was an extension at 72˚C for 1 minute. The reaction was kept at 4˚C until further processing. The amplified material was cleaned with Agencourt AMPure XP Reagent (Beckman Coulter Inc, Brea, CA, USA) according to the KAPA-provided protocol. Amplified sample libraries were quantified using Quant-iT™ PicoGreen dsDNA Reagent (Life Technologies, Carlsbad, CA, USA).

Liquid Phase Sequence Capture. Prior to hybridization, amplified sample libraries with unique barcoded adapters were combined in equal amounts into 1.1 µg pools for multiplex sequence capture. Exome sequence capture was performed with NimbleGen's SeqCap EZ Human Exome Library, either v3.0 with 64 Mb of exonic sequence targeted (DCEG Familial Exome), or Exome+UTR with 96Mb targeted (DCEG controls) (Roche NimbleGen, Inc., Madison, WI, USA). Prior to hybridization the following components were added to the 1.1 µg pooled sample library, 4 µL of NEXTflex HE Universal Oligo 1, 250 µM (5'-AATGATACGGCGACCACCGAGATCTACACTCTTTCCCTACACGACGCTCTTCCGATCT-3'), 40 µL total 25 µM NEXTflex INV-HE blocking oligos, equal volumes of each blocking oligo complementary to the barcodes in the pool (5'-CAAGCAGAAGACGGCATACGAGAT**X**GTGACT

GGAGTTCAGACGTGTGCTCTTCCGATCT/C3 Spacer/-3', where **X** is 8-bases of sequence specific to adapter barcode used for library construction), and 5 μL of 1 mg/mL COT-1 DNA (Invitrogen, Inc., Carlsbad, CA, USA). Samples were dried down by puncturing a hole in the plate seal and processing in an Eppendorf 5301 Vacuum Concentrator (Eppendorf, Hauppauge, NY, USA) set to 60˚C for approximately 1 hour. To each dried pool, 7.5 μL of NimbleGen Hybridization Buffer and 3.0 μL of NimbleGen Hybridization Component A were added, and placed in a heating block for 10 minutes at 95˚C. The mixture was then transferred to 4.5 μL of EZ Exome Probe Library and hybridized at 47˚C for 64 to 72 hours. Washing and recovery of captured DNA were performed as described in NimbleGen SeqCap EZ Library SR Protocol.

Post-hybridization LM-PCR. Pools of captured DNA were amplified by ligation-mediated PCR consisting of one reaction for each pool containing 20μl captured library DNA, 25 μL 2x KAPA HiFi HotStart ReadyMix, and 5μL 10x Library Amplification Primer Mix (includes two primers whose sequences are: 5'-AATGATACGGCGACCACCGA-3' and 5'-CAAGCAGAAGACGGCATACGA-3'). PCR cycling conditions were as follows: 98˚C for 45 seconds, followed by 8 cycles of 98˚C for 15 s, 60˚C for 30 s, 72˚C for 30 s. The last step was an extension at 72˚C for 1 minute. The reaction was kept at 4˚C until further processing. The amplified material was cleaned with Agencourt AMPure XP Reagent (Beckman Coulter Inc, Brea, CA, USA) according to NimbleGen SeqCap EZ Library SR Protocol. Pools of amplified captured DNA were then quantified via Kapa's Library Quantification Kit for Illumina (Kapa Biosystems, Woburn, MA, USA) on the LightCycler 480 (Roche, Indianapolis, IN, USA).

Exome sequencing. The human reference genome and the "known gene" transcript annotation were downloaded from the UCSC database (http://genome.ucsc.edu/), version hg19 (corresponding to Genome Reference Consortium assembly GRCh37). Sequencing reads were first trimmed using the Trimmomatic program (v0.32)[1], which marks all low-quality stretches (average quality score < Q15 in a 4-bp sliding window) and reports the longest high-quality stretch of each read. Only read pairs with both ends no shorter than 36 bp were used. Reads were then aligned to the hg19 reference genome using Novoalign

software (v3.00.05) (http://www.novocraft.com). Duplicate reads due to either optical or PCR artifacts were removed from further analysis using the MarkDuplicates module of the Picard software (v1.126) (http://picard.sourceforge.net/). Additionally, our analysis used only properly aligned read pairs, in the sense that the two ends of each pair must be mapped to the reference genome in complementary directions and must reflect a reasonable fragment length (300+/-100 bp). These high-quality alignments for each individual were further refined according to a local realignment strategy around known and novel sites of insertion and deletion polymorphisms using the RealignerTargetCreator and IndelRealigner modules from the Genome Analysis Toolkit[2] (GATK v3.1). Bam file level recalibration was also performed using BaseRecalibrator module from GATK.

Variant discovery and genotype calling of multi-allelic substitutions, insertions and deletions were performed on all individuals globally using the UnifiedGenotyper and HaplotypeCaller modules from Genome Analysis Toolkit (GATK v3.1) as well as the FreeBayes variant caller (v9.9.2). The Ensemble variant calling pipeline (v0.2.2 http://bcb.io/2013/02/06/an-automated-ensemble-method-for-combining-and-evaluating-genomic-variants-from-multiple-callers/) was then implemented to integrate analysis results from above three callers. Then Ensemble variant calling pipeline applies a machine learning algorithm called Support Vector Machine (SVM) to identify an optimal decision boundary based on the variant calling results out of multiple variant callers, with an aim to improve the caller's receiver operating characteristic – in other words, a more balanced decision between false positives and true positives.

In addition, insertions and deletions were left-aligned at both post-alignment (BAM) and post-variant-calling (VCF) levels using GATK's LeftAlignIndels and LeftAlignVariants modules, respectively. Annotation and variants dissemination (optional) were performed using our in-house custom software annotation pipeline. This pipeline adds different types of functional annotations that range from DNA level, to RNA level and to protein/histone level through integration of multiple public-domain applications including SnpEff/SnpSift (http://snpeff.sourceforge.net/) ANNOVAR[3]

(http://www.openbioinformatics.org/annovar/), etc. and public databases such as UCSC GoldenPath database (http://hgdownload.cse.ucsc.edu/goldenPath/hg19/database/), ESP6500 dataset from University of Washington's Exome Sequencing Project (http://evs.gs.washington.edu/EVS/), dbNSFP - database of human nonsynonymous SNPs and function predictions[4] (https://sites.google.com/site/jpopgen/dbNSFP), the Molecular Signatures Database - MSigDB (http://www.broadinstitute.org/gsea/msigdb/index.jsp), National Center for Biotechnology Information dbSNP database[5] build 137, and 1000 Genomes Project[6], etc.

Matching cases and controls using ethnicity-informative variation.

We imported Northern and Western European ancestry (CEU), African ancestry (YRI), Asian ancestry (ASA) (Han Chinese and Japanese) subjects from 1000 Genome (1KG) for the admixture analysis. For each of the ethnic groups, we imported 168808 SNPs overlapping to the dataset and filtered out monomorphic SNPs in any of the ethnic group and ended up with 37342 SNPs. We used these SNPs to estimate the ethnic components of the dataset. The estimation was made with an in-house imputation of the algorithm described in Pritchard et al. 2000[7].

The majority samples were estimated to be CEU. We excluded samples with CEU component < 0.80 and carried out the principal component analysis with the remaining samples.

Quality control, capture region matching and ACMG-59 genes. After controls and cases were matched, poor quality and contaminated samples were excluded from the dataset. Any variants that were flagged with our pipeline quality control metric (CScorefilter), had a read depth <10, ABHet <0.2 or > 0.8 or did not pass other quality control filters were excluded from the analysis. Since the capture kits differed between cases and controls, the capture regions were matched, primarily by excluding the untranslated region (UTRs) sequences in cases. All variants were further filtered using popmaxfreq < 0.01. Annotated variants were divided into cancer (*APC* (ENST00000257430), *BMPR1A* (ENST00000372037), *BRCA1* (ENST00000471181), *BRCA2* (ENST00000380152), *MEN1*(ENST00000337652), *MLH1*

(ENST00000231790), *MSH2* (ENST00000233146), *MSH6* (ENST00000234420), *MUTYH* (ENST00000450313), *NF2* (ENST00000338641), *PMS2* (ENST00000265849), *PTEN* (ENST00000371953), *RB1* (ENST00000267163), *RET* (ENST00000355710), *SDHB* (ENST00000375499), *SDHC* (ENST00000367975), *SDHD* (ENST00000375549), *SMAD4* (ENST00000342988), *STK11* (ENST00000326873), *TP53* (ENST00000269305), *TSC1* (ENST00000298552), *TSC2* (ENST00000219476), *VHL* (ENST00000256474), *WT1* (ENST00000332351)) and non-cancer (*ACTA2* (ENST00000224784), *ACTC1* (ENST00000290378), *APOB* (ENST00000233242), *ATP7B* (ENST00000242839), *CACNA1S* (ENST00000362061), *COL3A1* (ENST00000304636), *DSC2* (ENST00000280904), *DSG2* (ENST00000261590), *DSP* (ENST00000379802), *FBN1* (ENST00000316623|), *GLA* (ENST00000409170), *KCNH2* (ENST00000262186), *KCNQ1* (ENST00000155840), *LDLR* (ENST00000557933), *LMNA* (ENST00000368300), *MYBPC3* (ENST00000399249), *MYH11* (ENST00000396324), *MYH7* (ENST00000355349), *MYL2* (ENST00000228841), *MYL3* (ENST00000292327), *OTC* (ENST00000039007), *PCSK9* (ENST00000302118), *PRKAG2* (ENST00000287878), *RYR1* (ENST00000359596), *RYR2* (ENST00000360064), *SCN5A* (ENST00000333535), *SDHAF2* (ENST00000301761), *SMAD3* (ENST00000327367), *TGFBR1* (ENST00000552516), *TGFBR2* (ENST00000359013), *TMEM43* (ENST00000306077), *TNNI3* (ENST00000344887), *TNNT2* (ENST00000458432), *TPM1* (ENST00000357980)) genes from ACMG-59 gene list[8].

Supplemental Reference

1. Lohse M, Bolger AM, Nagel A, Fernie AR, Lunn JE, Stitt M, Usadel B: **RobiNA: a user-friendly, integrated software solution for RNA-Seq-based transcriptomics**. *Nucleic Acids Res* 2012, **40**(Web Server issue):W622-627.

2. DePristo MA, Banks E, Poplin R, Garimella KV, Maguire JR, Hartl C, Philippakis AA, del Angel G, Rivas MA, Hanna M *et al*: **A framework for variation discovery and**

genotyping using next-generation DNA sequencing data. *Nat Genet* 2011, **43**(5):491-498.

3. Wang K, Li M, Hakonarson H: **ANNOVAR: functional annotation of genetic variants from high-throughput sequencing data**. *Nucleic Acids Res* 2010, **38**(16):e164.
4. Liu X, Jian X, Boerwinkle E: **dbNSFP: a lightweight database of human nonsynonymous SNPs and their functional predictions**. *Hum Mutat* 2011, **32**(8):894-899.
5. Sherry ST, Ward MH, Kholodov M, Baker J, Phan L, Smigielski EM, Sirotkin K: **dbSNP: the NCBI database of genetic variation**. *Nucleic Acids Res* 2001, **29**(1):308-311.
6. Genomes Project C, Abecasis GR, Altshuler D, Auton A, Brooks LD, Durbin RM, Gibbs RA, Hurles ME, McVean GA: **A map of human genome variation from population-scale sequencing**. *Nature* 2010, **467**(7319):1061-1073.
7. Pritchard JK, Stephens M, Donnelly P: **Inference of population structure using multilocus genotype data**. *Genetics* 2000, **155**(2):945-959.
8. Kalia SS, Adelman K, Bale SJ, Chung WK, Eng C, Evans JP, Herman GE, Hufnagel SB, Klein TE, Korf BR *et al*: **Recommendations for reporting of secondary findings in clinical exome and genome sequencing, 2016 update (ACMG SF v2.0): a policy statement of the American College of Medical Genetics and Genomics**. *Genet Med* 2017, **19**(2):249-255.
