# Supplementary material for: Prevalence of pathogenic/likely pathogenic variants in the 24 cancer genes of the ACMG Secondary Findings v2.0 list in a large cancer cohort and ethnicity-matched controls
Source: Genome Med. 2018 Dec 24;10:99. doi: 10.1186/s13073-018-0607-5 (PMC6305568; doi:10.1186/s13073-018-0607-5)
Supplement: Supplementary file 2 — Figure S1. Population stratification of cancer cases and controls. Figure S2. Principal component analysis of cancer cases and controls. Table S1. Study names and predominant cancer types in DCEG Familial Exome cohort. (PDF 449 kb) [file 13073_2018_607_MOESM2_ESM.pdf]

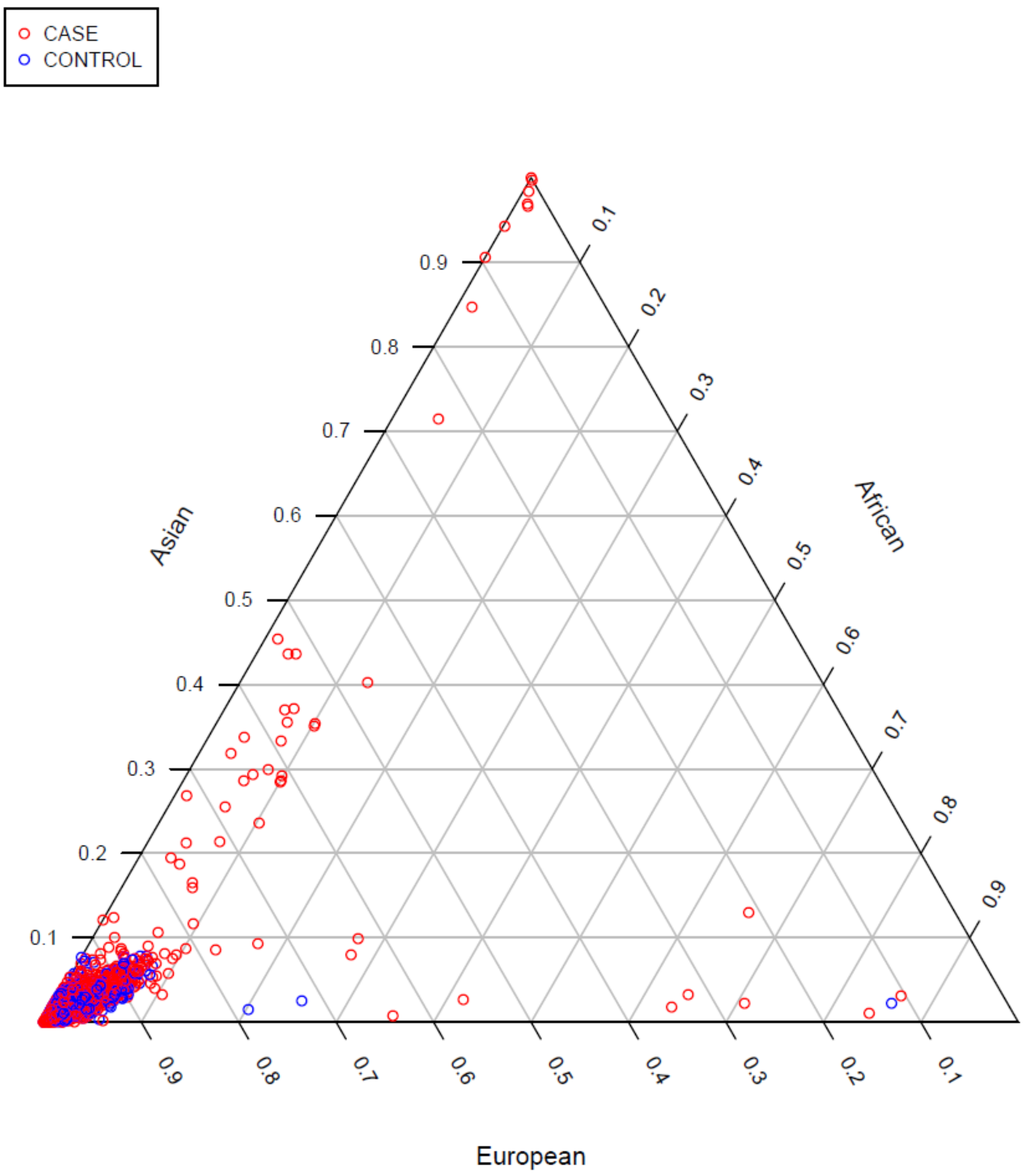

Fig S1: Population stratification of cancer cases and controls.
Cancer cases from the DCEG Familial Exome cohort (red, total n = 1388) and cancer-free controls (blue, total n = 1001) from two cohort studies (Prostate, Lung, Colorectal and Ovarian Cancer Screening Trial (PLCO) and the Cancer Prevention Study (CPSII) of the American Cancer Society) and one case-control study (Environment and Genes in Lung Cancer Etiology (EAGLE)). After filtering for CEU>0.8, there were 995 cases and 1339 controls.

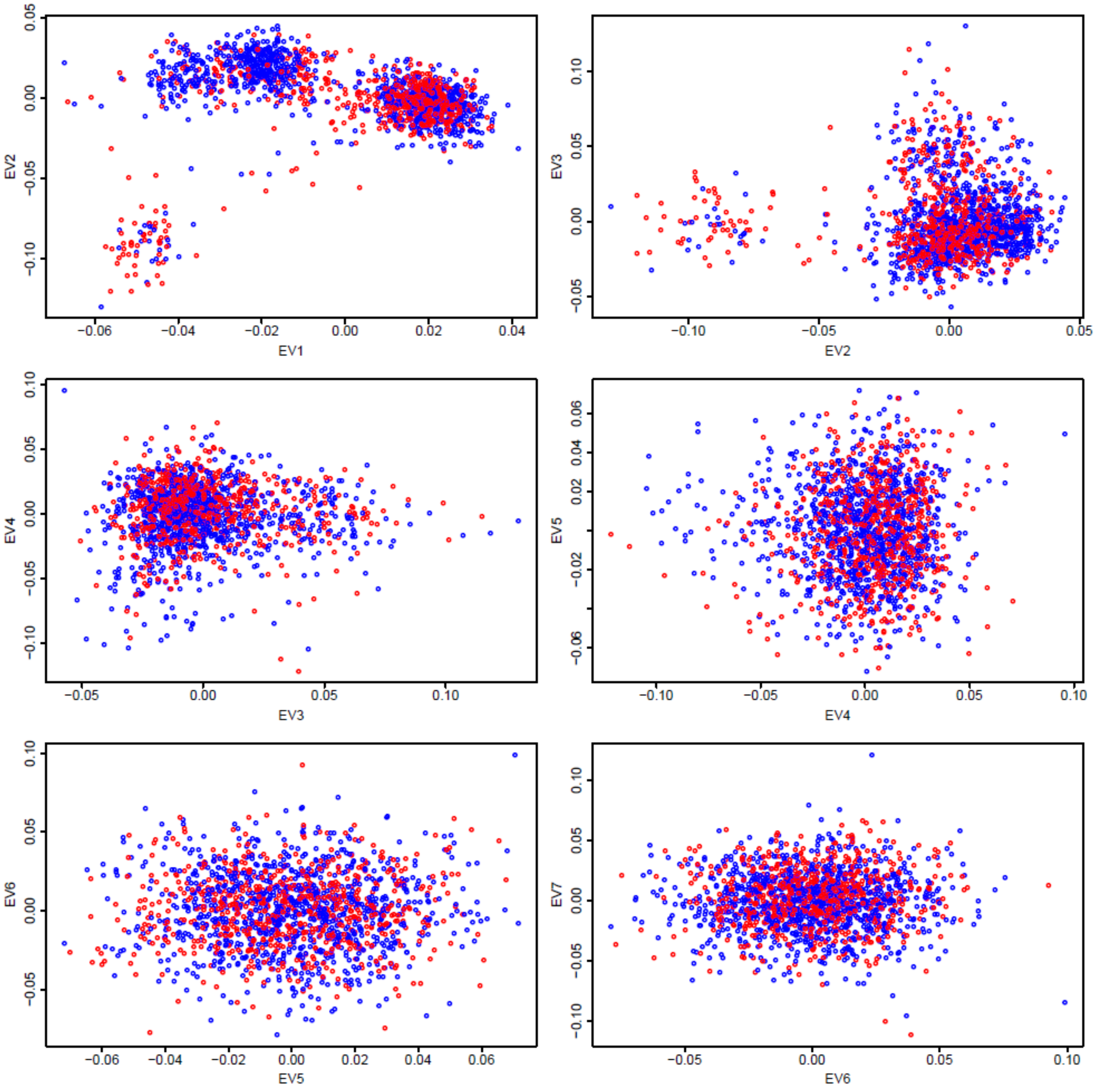

Fig S2: Principal component analysis of cancer cases and controls.
Principal component analysis of cancer cases (red) and controls (blue) with variation-derived ethnicity CEU > 0.8 matching.
Abbreviations: EV-eigen vector; CEU-Northern and Western European ancestry

Table S1. Study names and predominant cancer types in DCEG Familial Exome cohort. The "Sample Number" and "Percent of Total" reflect the numbers of samples in the study prior to the quality control process (n = 1388), not the total number of samples after quality control (n = 1173).

Study Name	Predominant Cancer Type	Sample Number	Percent of Total
Detection of genes predisposing to lymphoid malignancies	Leukemia and lymphoma	360	26
Gene discovery in familial breast/ovarian cancer through whole-exome sequencing	Breast and ovarian cancer	25	1.8
Analyzing Familial Bladder Cancer Families with Whole Exome Sequencing to Uncover Germline Allelic Variants That Confer Risk of Bladder Cancer	Bladder cancer	8	0.6
Identification of novel susceptibility genes for familial melanoma in Italian/American/Spanish melanoma families without known mutations using exome sequencing	Melanoma	441	32
Uncovering the role of germline mutations in familial testicular cancer through whole-exome sequencing	Testicular cancer	271	20
Gene discovery in Blackfan-Diamond anemia through whole-exome sequencing	Colon Adenocarcinoma; Osteosarcoma; Myelodysplastic syndrome; acute myeloid leukemia	52	3.7
Exome Sequencing in Dyskeratosis Congenita	Head and neck squamous cell carcinoma; Myelodysplastic syndrome; acute myeloid leukemia	55	4.0
Gene discovery in Fanconi anemia through whole-exome sequencing	Head and neck squamous cell carcinoma; acute myeloid leukemia	10	0.7
Exome Sequencing in Thrombocytopenia Absent Radii Syndrome	Acute leukemia	5	0.4
Gene discovery in Shwachman–Diamond syndrome through whole-exome sequencing	Myelodysplastic syndrome; Acute myeloid leukemia	7	0.5
Exome sequencing of *DICER1*-negative individuals with pleuropulmonary blastoma (PPB)	Pleuropulmonary blastoma	2	0.1

Gene discovery in familial myelodysplastic syndrome through whole-exome sequencing	Acute myeloid leukemia	2	0.1
Detection of genes predisposing to lung malignancies	Lung cancer	5	0.4
Exome Sequencing in Familial Diffuse Intrinsic Pontine Glioma (DIPG)	Diffuse intrinsic pontine glioma	5	0.4
Identification of novel susceptibility genes for familial and sporadic chordoma in patients without T duplications using exome sequencing	Chordoma	139	10
Cartilage-Hair Hypoplasia Syndrome	Lymphoma, basal cell carcinoma	1	0.07%
